# Peptide modulators of cell migration: Overview, applications and future development

**DOI:** 10.1016/j.drudis.2023.103554

**Published:** 2023-03-13

**Authors:** Jasmin Gattringer, Christian W. Gruber, Roland Hellinger

**Affiliations:** 1 Medical University of Vienna, Center for Physiology and Pharmacology, Schwarzspanierstrasse 17, A-1090 Vienna, Austria

**Keywords:** cell migration, peptide therapeutics, nature-derived peptides, stabilized peptide scaffolds, cyclotide, molecular grafting

## Abstract

Cell migration is a key physiological process in the development and homeostasis of multicellular organisms; errors in this complex system can trigger the development of cancer or inflammatory disorders. Therefore, modulating cell migration provides opportunities for drug discovery. Peptides are gaining importance on the global therapeutics market, given their unique properties compared with established small-molecule drugs or biologics. In this review, we identified over 470 peptides modulating cell migration and analyzed their characteristics. Over 95% of these peptides are in the discovery or preclinical stage, because the transition of peptide hits into drug leads often results in a bottleneck in the development process. We summarize chemical strategies in (pre-)clinical development to enhance drug-like properties of bioactive peptides.

## Introduction

Cell migration is an essential process of life. During early fetal development, stem cells migrate to defined sites of the embryo, initiating organ development or nervous system differentiation.^1^ Later in life, controlled migration of immune cells constitutes the natural basis for immune surveillance and immune function. Similarly, tissue renewal and wound repair are initiated by migration of epithelial cells (ECs), fibroblasts, or platelets.^2^ These are just a few examples; it is evident that cell migration is a complex multistep process. For more details on the principles of cell migration we refer the reader to other reviews^1,3,4^ of cancer^5,6^ and immune cell migration.^7–9^ Here, we provide a comprehensive overview of modulators of cell migration and highlight opportunities for future treatments of human diseases, including cancer and inflammatory disorders. In particular, we focus on existing peptide drugs and drug candidates in this field, discuss important examples of peptide cell migration modulators and review strategies for peptide drug development.

**Figure 1:**
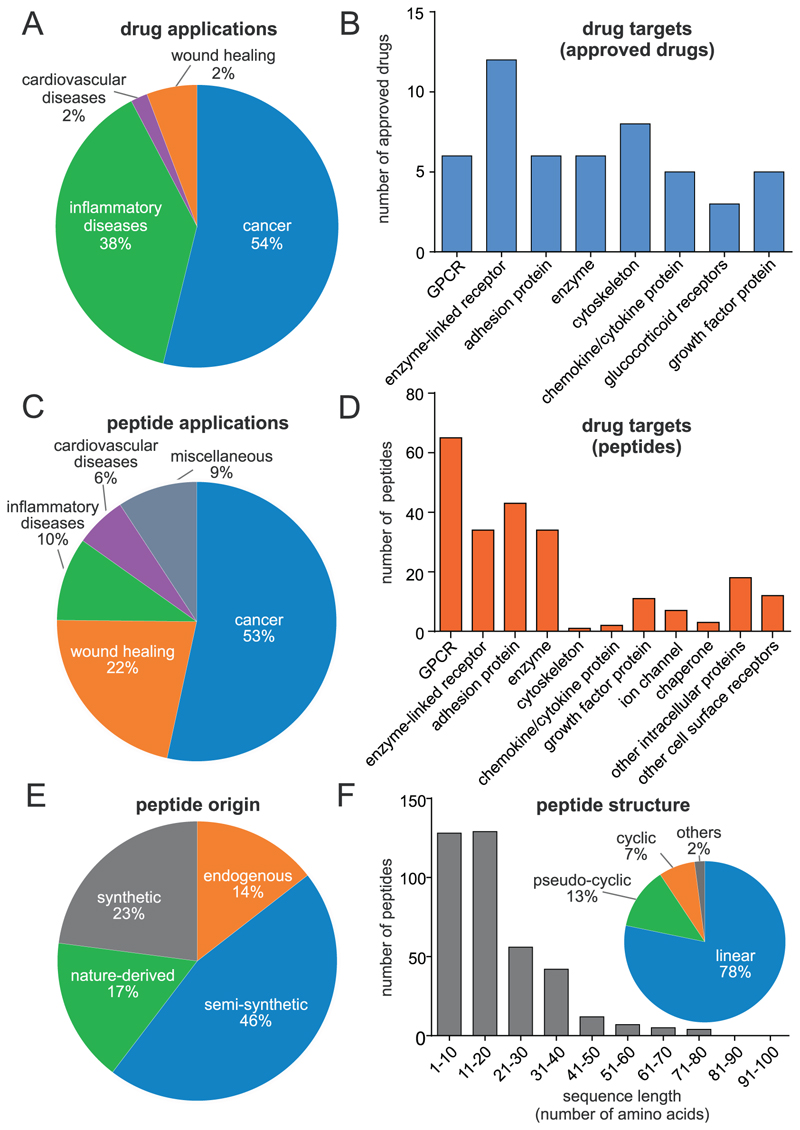
Analysis of approved drugs and of identified peptides with cell migration inhibitory activity. **(a)** From all US Food and Drug Administration (FDA)-approved drugs, we considered those therapeutics for which their cell migration inhibitory or stimulatory activity is reportedly essential for their described therapeutic effect. In total, we identified 51 approved drugs modulating cell migration, which are used for the treatment of cancer, inflammatory or cardiovascular diseases, and to promote wound healing. **(b)** The molecular drug targets of the approved drugs were identified to be G-protein-coupled receptors (GPCRs), enzyme-linked receptors, adhesion proteins, enzymes, cytoskeleton proteins, glucocorticoid receptors, cytokine/chemokine proteins, and growth factor proteins. **(c)** A literature search for peptides with modulatory activity of cell migration was performed, resulting in 479 curated peptides for further analysis. We included peptides in our analysis if their reported bioactivities related to cell migration indicated a relevance for a possible disease therapy; therefore, we excluded peptides that, for example, promoted cancer cell migration and metastasis or were proinflammatory peptides. In the case of hits derived from peptide library screens and combinatorial derivatives of peptides, only the most interesting (e.g., the hit with the best bioactivity) of the study was included in this analysis. Peptides with the integrin-binding RGD sequence were used in 136 studies and we included those peptides for which the RGD integrin binding motif was the primary moiety for activity. We identified the following fields of application for these peptides: cancer (53%), wound healing (22%), inflammatory diseases (10%), and cardiovascular disease (6%); fields with less than 5% were merged in a miscellaneous group (e.g., pulmonary disease or neurodegenerative diseases). Peptides with classification into two or more applications by independent studies were counted multiple times for this analysis. **(d)** We could identify the molecular targets of 232 peptides, which were classified as: GPCRs, enzyme-linked receptors, adhesion proteins, enzymes, cytoskeleton proteins, glucocorticoid receptors, cytokine/chemokine proteins, growth factor proteins, ion channels, chaperons, other cell surface receptors, and intracellular proteins, and a miscellaneous group for protein targets that did not fit into one of the classifications. **(e)** The source of origin of the identified peptides was categorized as either synthetic (synthetic), nature-derived (nature-derived), found as an endogenous peptide in humans (endogenous), or derived from any human endogenous peptide or protein sequence by synthetic modifications (semi-synthetic). **(f)** The peptide sequence length for 383 hits, for which the corresponding information was unequivocally available in the literature, was analyzed. Not included in the reported peptide length were any sequences added to the bioactive peptide, such as cell permeability or linker sequences. The insert shows further information about the peptide structure and chemistry, which were available for 378 peptides. We classified peptides into linear, cyclic peptides, pseudo-cyclic peptides (cyclized through disulfide bonds) other cyclic peptides (e.g., through a cyclic backbone), and other peptides (e.g., branched peptides, dendrimers, or stapled peptides).

Cell movement gone rogue can be the cause for the development and progression of various diseases; therefore, it is not surprising that the modulation or inhibition of cell migration is a promising therapeutic concept that has previously been utilized in drug development. Currently, there are 51 US Food and Drug Administration (FDA)-approved drug entities available as modulators of cell migration. These drugs cover a broad range of molecular targets, such as G-protein-coupled receptors (GPCRs), integrins or enzyme-linked receptors (Figure 1a,b; Table S1 in the supplemental information online). To date, small molecules (66%, 33) and monoclonal antibodies (20%, ten) dominate the field of migration modulators. By contrast, there are few peptides (8%, five) and recombinant or fusion proteins (6%, three). Given that cell migration has a pivotal role in cancer development and progression, neoangiogenesis, and metastasis, most of the approved drugs are in use for this indication.^3,10^ Furthermore, cell migration drugs are frequently applied for chronic inflammatory and autoimmune diseases, and cardiovascular disorders, and cell migration is crucial for wound-healing responses and tissue regeneration.^11^

## Peptide modulators of cell migration

Peptide therapeutics are an emerging drug class with growing numbers of regulatory approvals over the past few years and several hundreds to thousands of candidates in the discovery or the (pre)-clinical phase.^12^ However, despite the success of peptide drugs in therapeutic areas such as cardiovascular or metabolic diseases, only a few peptides have been approved to modulate cell migration. Mammalian target of rapamycin (mTOR) inhibitors, such as sirolimus, everolimus, and temsirolimus, the calcineurin inhibitor tacrolimus, as well as the antiplatelet drug eptifibatide are currently, to our knowledge, the only approved peptide drugs in this area. Nonetheless, the number of peptide modulators of cell migration advancing to preclinical or early clinical phases is increasing. In this review, we report on a literature search on the current status of peptide modulators for cell migration.

The literature search covered peer-reviewed publications listed in PubMed (https://www.ncbi.nlm.nih.gov/pubmed). Two search terms (‘peptide AND cell migration’), restricted to abstract and keywords of any publication, were used. Most publications in the field of cell migration were manually curated to remove duplicates and redundant hits as well as peptides without therapeutic value (e.g., stimulation of cancer cell migration/metastasis or proinflammatory peptides). This resulted in 840 publications, starting with the earliest identified publication from 1982, describing a total of 479 unique peptides for which cell migration modulatory properties were evident (Table S2 in the supplemental information online). The detailed analysis of the data is summarized in Figure 1c–f.

We analyzed the indication of use for each peptide (Figure 1c). Similarly to approved drugs, peptides also exhibited a strong focus on cancer drug development (53%, 243). Interestingly, wound healing and tissue regeneration (21%, 99) for example by stimulating fibroblast migration, are important applications for these peptides. Further identified indications were inflammatory, autoimmune diseases (10%, 44), cardiovascular disorders (6%, 27), and several diseases collectively grouped in ‘others’ (9%, 42). A molecular target was identified for 232 of these peptides, whereas, for others, only phenotypic effects have been described. Membrane-bound proteins, such as integrins, GPCRs, or enzyme-linked receptors, are important targets for peptides, whereas few examples were found for intracellular protein targets (Figure 1d).

Next, we focused on the origin of the reported peptides for migration inhibition. Besides endogenous human peptide modulators of cell migration (65 peptides; 14%), such as the epidermal growth factor or human beta defensins, many (46%, 206) of the identified molecules were structurally related or chemically derived from human endogenous proteins or peptides (semi-synthetic peptides). In addition, 17% (75 peptides) corresponded to peptide natural products derived from plants, bacteria, fungi, invertebrate, or vertebrates, denoted as nature-derived peptides. Synthetically derived peptides, for example through screening synthetic combinatorial peptide libraries (e.g., phage display technology and similar to provide hits through affinity maturation), are another source for peptides (23%, 103 peptides) (Figure 1e). For 383 of the described peptides, further information, such as amino acid sequence, peptide length, or structural information (e.g., cyclization or disulfide bonds), could be obtained from the original published articles. Interestingly, the majority of the described peptides were short, linear peptides (78%, 295); for instance, more than half of the peptides had less than 20 residues (Figure 1f). The remaining peptides were pseudo-cyclic peptides cyclized through disulfide bonds (13%, 48), other cyclic peptides (7%, 27) as well as dendrimers or dimers (2%, eight).

Below, we discuss examples of peptide drug candidates in different developmental stages that, in our opinion, hold great promise for future applications in cancer, wound healing, inflammatory disorders, and cardiovascular diseases.

## Peptides targeting cancer cell migration

Cell migration is a major factor contributing to cancer progression. Inhibiting cancer cell migration and invasion of healthy tissues can prevent the development of secondary tumors. In addition, cancer cells have a high energy demand and develop mechanisms to induce vascularization to promote their energy supply.^5,13^ Besides already established drug targets, such as the mTOR protein complex, numerous possible targets have been exploited aiming to develop peptide anticancer therapeutics. In our literature search, we identified several possible targets of peptides, including GPCR, adhesion proteins, and enzymes, which we discuss in more detail below.

### GPCRs

GPCRs are drug targets for approximately one-third of all approved drugs. Numerous peptide migration inhibitors target chemokine receptors, and class B GPCRs (e.g., formyl peptide, neuropeptide Y, relaxin, kisspeptin, or apelin receptors). For example, CXC-motif chemokine receptor 4 (CXCR4) is a promising target for migration inhibition.^14,15^ It is overexpressed in various cancers, including multiple myeloma, breast and prostate cancer, and has been associated with cancer metastasis along with poor prognosis for patients.^16,17^ The approved small-molecule CXCR4 antagonist plerixafor is used in combination with granulocyte colony-stimulating factor (G-CFS) to mobilize hematopoietic stem cell migration from the bone marrow into the periphery before autologous hematopoietic stem cell transplantation.^18^ The endogenous CXCR4 ligand stromal derived factor 1 (SDF1; CXCL12) has been used as a template for the development of peptide antagonists. Furthermore, a CXCR4 antagonist peptide called EPI-X4, which was identified as a proteolytic fragment from an albumin precursor protein, inhibits cancer cell migration and stimulates the migration of hematopoietic stem cells.^19^ The nature-derived peptide motixafortide, isolated from a horseshoe crab protein, is a 14-mer cyclic CXCR4 antagonist (BL-8040).^20^ It is being investigated in multiple clinical trials for the treatment of leukemia and solid tumors. A phase II trial for the treatment of acute myeloma is ongoing (NCT02502968). Importantly, a phase III study of motixafortide in autologous hematopoietic transplantation in patients with myeloma in combination with G-CSF reached its designated primary and secondary endpoints.^21^ These results hold promise for future regulatory approval of this peptide.

### Adhesion proteins

Adhesion molecules have a major role in cell–cell and cell–extracellular matrix interactions and, therefore, are crucial for the ability of cells to migrate.^22,23^ For instance, the heterodimeric integrin, selectin, or cadherin families, are important examples of such adhesion proteins. Several peptide migration inhibitors have been explored in cancer drug development because cell–cell and cell–matrix proteins, such as CD44, different integrins, and cell surface proteins are recognized as interesting anticancer drug targets. The cyclic peptide cilengitide cyclo-(Arg-Gly-Asp-DPhe-NmetVal), is a specific inhibitor of αvβ3, α5β1, and αvβ5 integrins, which are overexpressed in glioblastoma and involved in neoangiogenesis.^24^ During the 1990s, pioneering preclinical developmental work led to the synthetic cyclic cilengitide, which had a 100–1000-fold increased activity compared with the prototype peptide and importantly increased integrin selectivity.^25^ Therefore, cilengitide was considered a promising anticancer drug with very little toxicity and excellent tolerance. Among others, it was tested in a Phase III clinical study to treat glioblastoma.^26^ However, the translation to patients appeared difficult, because study outcomes did not indicate significance in overall survival compared with standard treatment.^26,27^ A plasma half-life of ~4 h for the circular peptides was achieved in patients. The study design used a twice-weekly intravenous administration in a high-dose regime because the drug candidate was well tolerated in patients. However, limited transport into the central nervous system was an Achilles heel of the cyclic peptide regarding its application to treat brain glioblastoma.^28^ Pharmacokinetic data showed that only 0.01% of cilengitide reached the cerebrospinal fluid compared with plasma levels.^29^ Recently, cilengitide and other RGD peptides experienced a revival as antiviral drug candidates during the severe acute respiratory syndrome-coronavirus 19 (SARS-CoV-19) pandemic. They were shown to block viral cell entry because the viral S-protein, harboring a RGD sequence, binds to endothelial α5β1 and αVβ3 integrin as co-receptors.^30^

The surface adhesion protein CD44 promotes migration and invasion processes relevant for metastasis and has been considered as an anticancer target. For example, the linear 23-mer anti-angiogenic peptide ALM201 is a semi-synthetic molecule derived from the human protein FK506-binding protein-like. It inhibits endothelial cell migration and angiogenesis as well as cancer cell migration and metastases via CD44-dependent mechanisms.^31,32^ A recently completed Phase I clinical trial in patients with ovarian cancer and other solid tumors indicated a good safety profile, supporting further clinical studies.^33^ ALM201 was awarded orphan drug status by the FDA as a clinical candidate for the development of new drugs toward high-grade serious ovarian carcinomas.^32^

### Enzymes

A variety of enzymes are involved in cell migration, including proteases, kinases, and other enzymes, which are crucial players in intracellular signaling networks.^34^ For example, matrix metalloproteases (MMPs), which break down extracellular matrix facilitating cell migration, have been investigated in several clinical studies for cancer treatment. However, these studies failed to develop a mature drug for various reasons, including specificity difficulties of the tested small-molecule compounds being able to target single MMPs.^35,36^ Peptides targeting MMPs could provide an interesting alternative to small molecules, especially because they are considered very specific for their target,^37^ thereby reducing unwanted off-target effects of multi-MMP inhibitors. For instance, a study utilized a bicyclic-peptide phage-display library to mature the affinity of probes toward MMP2. Alanine and D-amino acid scans were performed to identify the binding site in the peptides, which was rationally designed to incorporate a hydroxamate zinc-chelating moiety to further improve affinity for the target protease. The resulting peptide was the first synthetic MMP2 inhibitor, with K_i_ ~ 1.9 nM *in vitro* and a good selectivity profile over 11 MMPs, outperforming small-molecule inhibitors.^38^.

## Peptides targeting immune cell migration

Immune cell migration is an important driver in inflammatory and autoimmune diseases. Thus, inhibiting pathological immune cell migration can be useful in disease therapy.^39^ Adhesion proteins, such as integrins, are targeted by approved drugs to inhibit immune cell migration for the treatment of autoimmune diseases. Other targets include the GPCR family of chemokine receptors, which guide immune cells toward gradients of their chemical cues to inflammatory sites.^40^ In addition, we identified enzymes (e.g., Janus kinase) or kinase-linked receptors (e.g., insulin-like growth factor receptor) as targets for peptides modulating immune cell migration. We highlight the GPCR family of sphingosine-1-phosphate receptors 1–5 (S1PR1–5) with their endogenous lipid mediator sphingosine-1-phosphate (S1P) as an important example of a promising target for peptides modulating immune cell migration. S1PRs regulate fundamental biological processes and have important roles in endothelial cell function and immune cell homeostasis. In T cells, which express S1PR1 and S1PR4, and other immune cells, these receptors are crucial checkpoints for cell trafficking.^41,42^ For example, the S1PR1 system guides T lymphocyte migration along a gradient of S1P, out of the lymph node and into blood circulation.^41^ The approval of fingolimod (FTY-720), a S1PR1 and S1PR3-5 ligand, by the FDA in 2010 marked a milestone in the treatment of relapsing-remitting multiple sclerosis. Through constant receptor internalization and degradation, FTY-720 depletes lymphocytes from cell surface-exposed S1PR, and this functional antagonism results in peripheral lymphopenia as well as lymphocyte retention in lymph nodes. To date, several drugs (e.g., siponimod) have been approved with similar indications that target this receptor signaling system.^42^ The peptide inhibitor of transendothelial migration (pepitem) is a small endogenous peptide that modulates S1PR signaling to inhibit transendothelial immune cell migration. Adiponectin-stimulated B cells have the capacity to release this peptide into the extracellular environment. Pepitem can stimulate endothelial cells to release S1P into the endothelial cell microenvironment after activation of an as yet not fully established mechanism that stimulates the activity of sphingosine kinase 1. The induced S1P signaling on adherent T lymphocytes unfolds its activity on target genes downstream of the signaling cascade. As a result, the transendothelial migration of T lymphocytes is reduced whereas their adhesion to endothelial cells is not altered by pepitem. The inhibitor was used to reduce cell infiltration into diseased tissues in models of T lymphocyte-dependent inflammatory or autoimmune diseases, such as glomerulonephritis^43^ and in a model of systemic lupus erythematosus.^44^ However, the overall low stability and short half-life in biological fluids of the prototypic pepitem molecule are limitations and restricts further therapeutic applications of this peptide.

## Peptide modulators of migration in wound healing

Cell migration also has a crucial role in healing wounds, tissue repair, and regeneration. For example, keratinocytes, fibroblasts, and EC migration is observed in wound repair. In addition, endothelial cell migration for vascularization promotes tissue renewal.^45^ Our literature search identified numerous peptides modulating cell migration to support wound healing, indicating promising new treatment options. Molecular targets for these peptides were found not only to be GPCRs (e.g., formyl peptide receptors), but also kinase-linked receptors (e.g., epidermal growth factor receptor), enzymes (e.g., MMPs) or adhesion proteins (e.g., integrins). One example is the endogenous antimicrobial peptide LL-37, belonging to the cathelicidin family. The peptide induces endothelial cell migration, thereby promoting vascularization as well as stimulation of migration of ECs, fibroblasts, and keratinocytes.^46^ Phase II clinical trials concluded that LL-37 improves wound healing in patients with hard-to-heal venous leg ulcers.^47,48^ Other examples are the endogenous histatins, which are important wound-healing peptides found in human saliva,^49^ the annexin A1-derived peptide Ac2-26,^50^ or the frog-skin derived peptide esculentin 1-21,^51^ which were shown to enhance wound-healing responses in preclinical studies.

## Peptide modulators of migration in cardiovascular diseases

The literature search provided peptides with application in the cardiovascular disease field. For example, in atherosclerosis development, smooth muscle cell migration and infiltration of immune cells participate in atherosclerotic plaque formation. Therefore, modulation of these cell migratory processes can prevent development of atherosclerosis.^52^ The literature search identified GPCRs (e.g., glucagon-like peptide 1 receptor), adhesion proteins (e.g., N-cadherin), kinase-linked receptors (e.g., platelet-derived growth factor) as well as enzymes (e.g., Janus kinase) as possible peptide targets in this area. Eptifibatide, an arginine-glycine-aspartic acid (RGD) mimetic, is approved as an anticoagulation medication. It ameliorates platelet aggregation by blocking the platelet integrin glycoprotein IIb/III receptor and the drug is used to prevent thrombotic events in myocardial infarction.^53^ The RGD tripeptide and other integrin-binding motifs, such as leucine-aspartic acid-valine-proline (LDVP), are well known for specific binding to integrin subtypes, which can be exploited in drug development.^54^ Interestingly, natural peptides, including many venom peptides, contain integrin-binding motifs. Their versatility makes them interesting starting points for drug development. For example, snake venom-derived disintegrins (eptifibatide was developed from the disintegrin barbourin) are small cysteine-rich integrin antagonists that inhibit the migration of various cell types. Several candidates have been studied for use in cardiovascular disease related to platelet aggregation^55^ and inhibition of angiogenesis,^56^ as well as in the cancer field.^57^

## Future aspects

**Figure 2:**
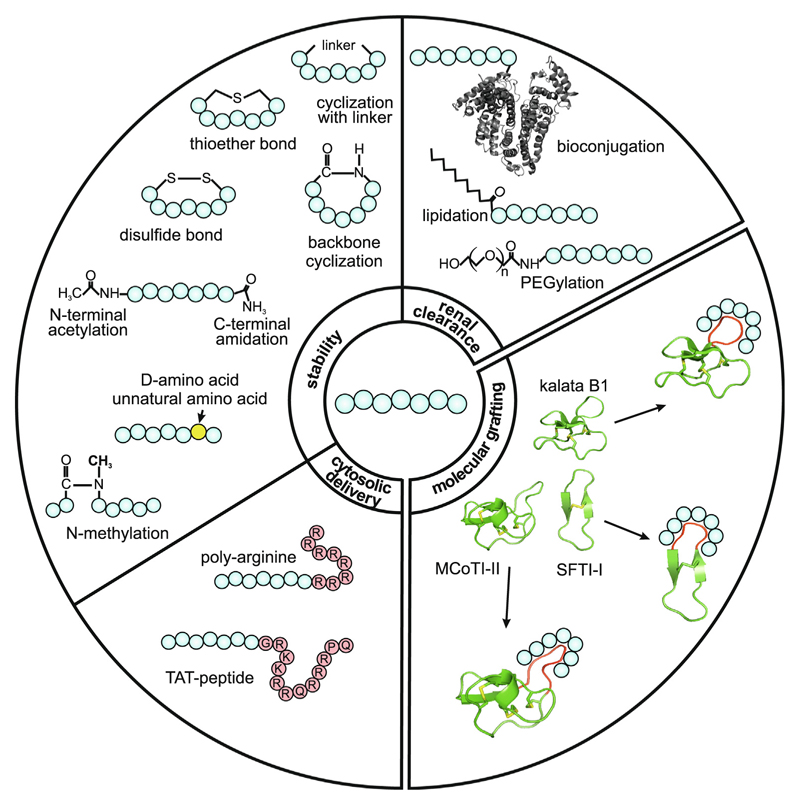
Overview of chemical strategies to improve the drug-like properties of peptides. Medicinal chemists have established approaches in peptide drug development to overcome several weaknesses of peptides for therapeutic use. For example, manifold strategies have been elaborated to improve the proteolytic instability of peptides in biological fluids. These include: protection of vulnerable N and C termini (acetylation or amidation, respectively) as well as cyclization through thioether bonds, various linker moieties, head-to-tail ligation (backbone-cyclization), or through disulfide bonds. D-amino acids or unnatural amino acids as well as backbone modifications, such as *N*-methylation, provide further protection from decomposition by endopeptidases. To reduce the fast renal clearance and short circulation times of peptides, bioconjugation to larger proteins, such as albumin [Protein Data Bank (PDB) ID: 1uor), lipidation by conjugation to fatty acid chains, or PEGylation of peptides are well-established methods. For delivery to intracellular sites and to overcome biological barriers, conjugation to cell-penetrating peptide sequences, such as the TAT peptide or the poly-arginine sequence (R8), has been used. A future trend in peptide drug design is molecular grafting through engineering natural stabilized peptide scaffolds to accommodate a bioactive sequence. Three prototypic plant-derived peptide scaffolds, the cyclotide kalata B1 (PDB ID: 1nb1), the *Momordica cochinchinensis* trypsin inhibitor 1 (MCoTI-II; PDB ID:1ib9) as well as the sunflower trypsin inhibitor 1 (SFTI-1; PDB ID: 1jbl) are highlighted because they have been used to prepare cell migration-inhibiting probes.

Many peptides modulating cell migration (Table S2 in the supplemental information online) have been discovered and some have been or are being investigated in (pre-)clinical studies, but only a few candidates have progressed into advanced clinical development stages. Peptides often lack important properties needed for therapeutic application and drug development. For example, they have very low proteolytic stability and short circulation times, rendering them unsuitable for *in vivo* application. Although few fast-acting peptide drugs, such as oxytocin or insulin derivatives, exist for which a limited stability is pivotal for their safe application in human therapy, higher biological stability and half-lives are usually mandatory for peptide therapeutics. Therefore, it might be beneficial to improve the stability of peptide migration inhibitors in biological fluids and/or circumvent their usually fast renal clearance for therapeutic applications. Here, we summarize chemical strategies in peptide drug development to improve stability in biological fluids and *in vivo* circulation time by decreasing renal clearance and cytosolic delivery (Figure 2).

### Biological stability of peptides

The half-life of a drug determines its residence time in the body. A short half-life can be compensated for by dosing interval and dosage, with limitations arising regarding costs and safety. Approximately 80 peptide therapeutics have received regulatory approval^58^ and are important examples of how medicinal chemists have developed methodologies to effectively increase peptide stability from minutes to several hours or even longer. The plasma half-lives of approved peptide therapeutics are significantly enhanced compared with endogenous peptides. Peptide chemistry has used a plethora of tools to prolong the stability of peptide therapeutics. Early attempts in peptide research aimed to stabilize bioactive peptides against degradation and inactivation by blood or tissue proteases by using chemical modifications (Figure 2). For instance, the C and N termini of a peptide are especially vulnerable sites for degradation by fast-acting low substrate-specificity exopeptidases. The N-terminal amine group can be acetylated or a pyroglutamate group can be installed as protection. Amidation of the C-terminal carboxyl group reduces carboxypeptidase cleavage. Similarly, N-to-C-terminal cyclization as well as ring closure with linker moieties, (e.g., thioether bridge or 2,3-diaminopropionic acid) have been explored to address proteolytic weakness of peptides. Most approved drugs were further developmentally matured to improve *in vivo* properties. To achieve this, any identified vulnerable sites for endopeptidase cleavages in the backbone of the peptide are made resistant by mutagenesis using unnatural L-amino acids or D-analogs.^12^ Furthermore, backbone modifications can be achieved by using β-amino acids, peptoids, or *N*-methyl residues.^58^ To make a peptide stabile when exposed to the proteome machinery, a retro-enantio approach can be utilized, which reverses the sequence of a peptide using only D-amino acid analogs.^59^ There are further promising strategies for the chemical design of peptide therapeutics to increase stability, such as stabilization of α-helix, β-sheet, or β-strand secondary structures using crosslinking or helix stapling. These have been reviewed further elsewhere.^12,58,60^

### Improving renal clearance

Peptides often have fast renal clearance, which limits drug exposure in a therapeutic concentration over time. Plasma circulation can be improved by linking peptides to biomolecules (Figure 2), with the aim to enhance circulation time through renal clearance escape. Lipid conjugated peptides, for instance to C_18_-fatty acid chains, allows for serum albumin binding to reduce renal filtration of the molecule. Similar effects are achieved with peptide PEGylation.^60^ Bioconjugation strategies targeting the immunoglobulin G Fc-domain, which binds to the neonatal Fc receptor or to carrier proteins, such as serum albumin, have similar effects on the peptide drug circulation residence time.^12,58^

### Cytosolic delivery of peptides

Many peptides target extracellular proteins; thus, to reach intracellular targets, they need to cross the cell membrane. However, peptides typically only have limited ability to cross this biological barrier. To tailor a peptide for intracellular delivery, conjugation to a cell-penetrating peptide sequence enables the delivery of bioactive peptides to intracellular targets. Several cell-penetrating peptides are used in research, such as penetratin, poly-arginine, transportan, and the well-studied prototype TAT peptide^61^ (Figure 2).

## Concluding remarks

This review provides a comprehensive overview of peptides for modulation of cell migration from the discovery stage to (pre-) clinical studies, as well as for future therapeutic applications. Our analysis shows that peptides are valuable sources for migration modulators and that there is already a plethora of peptides showing promising bioactivity on cell migration. Peptide therapeutics are on the rise in drug development and peptides offer a great starting point, even though their instability or limited circulation time are challenges. We have discussed medicinal chemistry strategies to tackle some of these difficulties, such as protection from proteolytic degradation or bioconjugations to extend blood circulation, as well as the use of cell-penetrating peptides to improve cytosolic delivery. Further limitations of peptidic drug candidates are their very low bioavailability and difficulties in applying them via the oral route of administration.

These issues require further research and developmental work. For instance, a trend in peptide research utilized to generate peptides with drug-like properties is the use of natural stabilized scaffolds for the design of novel drug candidates (Figure 2). The ‘molecular grafting’ approach has been used many times, including for peptide probes targeting cell migration, the design of probes with remarkable stability, and to promote further drug-like properties. Box 1 summarizes this methodology in general and discusses its contributions to the field of peptides modulating cell migration. Thus, combining the strategies presented here shows great promise for peptide drug development. Concise peptide design and development through chemical modifications as well as the use of peptide scaffolds with drug-like properties for molecular grafting in one approach shows great potential for drug development success. For the migration modulatory drug field, we predict the growing significance of peptide therapeutics with more candidates advancing toward clinical studies and therapeutic applications in the near future.

## Supplementary Material

Supplementary data to this article can be found online at https://doi.org/10.1016/j.drudis.2023.103554.

Supplementary data 1

Supplementary data 2

## Data Availability

No data was used for the research described in the article.

## Box 1 Molecular grafting as a trend in peptide science

Molecular grafting is a promising trend in peptide drug development and the design of novel probes. The approach is used to engineer peptide therapeutics with drug-like properties. Conformationally constrained and highly stable peptides are utilized as scaffolds that become ‘fused’ with bioactive epitopes^62^ (Figure 2 in the main text). After synthesis of the probes and verification of their conformational integrity, the grafted peptides are selected based on their bioactivity. The resulting molecules combine the advantages of increased stability provided by the peptide scaffold and functionalization provided by the bioactive epitope. Several nature-derived peptides have been explored for this application, including cyclic cysteine knot peptides,^60,62^ lasso peptide,^12,62^ cyclic sunflower trypsin inhibitor 1,^63^ and h-defensins.^64^ The approach was used to prepare new probes targeting GPCRs,^63^ integrins,^64^ ion channels,^65^ and other cell-surface receptors such, as vascular endothelial growth factor receptors (VEGFRs),^66^ enzymes,^67^ or protein–protein interactions.^68^ Interestingly, molecular grafting also supported the development of peptide therapeutics with additional drug-like properties, such as cell penetration^69^ and oral bioavailability to induce therapeutic effects in animal models.^70^ However, the size of the bioactive epitope that can be grafted is limited by the structural or steric needs of the scaffold. Therefore, short sequences are more amenable for molecular grafting compared with large peptides. The analysis presented in Figure 1f (in the main text) shows that most peptide migration inhibitors are smaller than a 20-mer and are linear sequences; thus, their overall stability in the face of proteolysis in biological fluids is weak. Therefore, they are suitable starting points as bioactive epitopes for molecular grafting with the aim to improve peptide stability. Indeed, the molecular grafting strategy has been utilized in the field of migration inhibitory peptides. Here, we highlight several candidates with promising activity and increased stability.

The linear antiangiogenic peptide P15 decomposed within 2 h in human serum. Integration of the P15 sequence into the backbone-cyclized trypsin inhibitor peptide Momordica cochinchinensis trypsin inhibitor-II (MCoTI-II) not only significantly enhanced peptide stability to 8 h, but also improved the inhibitory properties on cell migration of human umbilical vein endothelial cells (HUVECs).^68^ Similarly, neuropilin receptor (NRP)-1 and -2 couple with various receptor tyrosine kinases, including several growth factors receptors. NRPs are highly expressed in cancer cells and promote cell growth. The kalata B1 peptide was used as template for a kalata B1-based peptide phage display library to mature affinity and antagonistic activity toward NRP1 and NRP2. The resulting cyclic probes showed nanomolar affinity to both receptors and inhibited HUVEC migration. The cell viability was not affected by the probes up to micromolar concentrations, which highlights the applicability of this method.^71^

Human thrombospondin-1 (TSP1) is an endogenous inhibitor of angiogenesis. A bioactive heptapeptide derived from the thrombospondin sequence inhibited angiogenesis by interacting with the scavenger receptor CD36. However, TSP1 degrades within 4 h in human serum. Grafting of the bioactive sequence either in MCoTI-II or in the sunflower trypsin inhibitor 1 scaffold not only resulted in significant improvement of stability of the peptide over 24 h, but also increased its activity to inhibit HUVEC migration.^72^

In summary, molecular grafting is a promising methodology to augment the stability of peptide therapeutics. There is an increasing number of publications using this strategy and we have highlighted examples demonstrating its feasibility and applicability for peptide drug design in the migration inhibitor field. We predict that molecular grafting will be added to the chemical toolbox for peptide drug development and that utilizing this method has the potential to develop valuable peptide drugs in the future.
