# Supplementary material for: Peptide modulators of cell migration: Overview, applications and future development
Source: Drug Discov Today. Author manuscript; Available in PMC 2024 May 2. (PMC7615922; doi:10.1016/j.drudis.2023.103554)
Supplement: Supplementary data 1 [file EMS195753-supplement-Supplementary_data_1.docx]

**Table 1. Approved drugs modulating cell migration.**

| Name | Trade name | Therapeutic area | Indications | Molecular target | Drug molecule type | Approval |
| --- | --- | --- | --- | --- | --- | --- |
| Fingolimod | Gilenya | autoimmune/inflammatory disease | multiple sclerosis | S1PR 1,3-5 | small molecule | 2011 (EMA) 2010 (FDA) |
| Siponimod | Mayzent | autoimmune/inflammatory disease | multiple sclerosis | S1PR1, S1PR5 | small molecule | 2019 (FDA) 2020 (EMA) |
| Ponesimod | Ponvory | autoimmune/inflammatory disease | multiple sclerosis | S1PR1 | small molecule | 2021 (FDA and EMA) |
| Ozanimod | Zeposia | autoimmune/inflammatory disease | multiple sclerosis ulcerative colitis | S1PR1, S1PR5 | small molecule | 2020 (FDA and EMA) |
| Natalizumab | Tysabri | autoimmune/inflammatory disease | multiple sclerosis | α4b1 and α4b7 integrin | monoclonal antibody | 2006 (FDA and EMA)* |
| Vedolizumab | Entyvio | autoimmune/inflammatory disease | ulcerative colitis, Crohn's disease | α4β7 integrin | monoclonal antibody | 2014 (FDA and EMA) |
| Lifitegrast | XIIDRA | autoimmune/inflammatory disease | dry eye disease | integrin lymphocyte function-associated antigen-1 | small molecule | 2016 (FDA) no EMA approval |
| Plerixafor | Mozobil | cancer | hematopoietic stem cell transplantation | CXCR4 | small molecule | 2009 (EMA) 2008 (FDA) |
| Bevacicumab | Avastin | cancer | non-small-cell lung carcinoma renal cell carcinoma breast, ovarian, colorectal, peritoneal, cervical neoplasms and fallopian tube neoplasms | VEGF | monoclonal antibody | 2005 (EMA) 2004 (FDA) |
| Aflibercept | Eylea | cancer | wet macular degeneration macular edema diabetes complications | VEGF-A, PIGF | fusion protein | 2012 (EMA) 2011 (FDA) |
| Ramucirumab | Cyramza | cancer | stomach neoplasms metastatic colorectal cancer non-small cell lung cancer hepatocellular carcinoma | VEGFR2 | monoclonal antibody | 2014 (FDA and EMA) |
| Sorafenib | *Nexavar* | cancer | hepatocellular and renal cell carcinoma | CRAF, BRAF, KIT, FLT-3, VEGFR-2, VEGFR-3, and PDGFR-ß | small molecule | 2006 (EMA) 2005 (FDA) |
| Sunitinib | Sutent | cancer | gastrointestinal stromal tumors renal cell carcinoma neuroendocrine tumors | PDGFRα and PDGFRβ, VEGFR1-3, KIT, FLT3, CSF-1R, RET | small molecule | 2006 (FDA and EMA) |
| Regorafenib | Stivarga | cancer | colorectal cancer gastrointestinal stromal tumours hepatocellular carcinoma | VEGFR1-3, TIE2, KIT, RET, RAF-1, BRAF, BRAFV600E,PDGFR, FGFR, CSF1R | small molecule | 2013 (EMA) 2012 (FDA) |
| Pazopanib | Votrient | cancer | renal cell carcinoma soft-tissue sarcoma | VEGFR1-3, PDGFR-α,–β, c-KIT | small molecule | 2010 (EMA) 2009 (FDA) |
| Vandetanib | Caprelsa | cancer | thyroid neoplasms | VEGFR2-3, EGFR, RET, BRK, TIE2, members of EPH and SRC family of kinases | small molecule | 2012 (EMA) 2011 (FDA) |
| Lenvatinib | Lenvima | cancer | differentiated thyroid, hepatocellular and endometrial carcinoma | VEGFR1-3, FGFR1-4, PDGF, PDGFRα, KIT, RET | small molecule | 2015 (FDA and EMA) |
| Cabozantinib | Cometriq | cancer | thyroid, renal cell and hepatocellular carcinoma | f MET, VEGFR, RET, GAS6 receptor, KIT, FLT3 | small molecule | 2014 (EMA) 2012 (FDA) |
| Ranibizumab | Lucentis | cancer | wet macular degeneration macular edema degenerative myopia diabetic retinopathy | VEGF-A | monoclonal antibody | 2007 (EMA) 2006 (FDA) |
| Becaplermin | Regranex | wound healing | diabetic neuropathic ulcers | PDGFR | recombinant protein | 1997 (FDA) no EMA approval (withdrawn 2012) |
| Paclitaxel | taxol | cancer | breast cancer pancreatic cancer non-small cell lung cancer | tubulin | small molecule | 1992 (FDA)  before 1995 in EU* |
| Cabazitaxel | Jevtana | cancer | prostate cancer | tubulin | small molecule | 2011 (EMA) 2010 (FDA) |
| Docetaxel | Taxotere | cancer | head and neck neoplasms non-small-cell lung cancer prostate, stomach and breast cancer adenocarcinoma | tubulin | small molecule | 1995 (EMA) 1996 (FDA) |
| Eribulin | Halaven | cancer | breast cancer liposarcoma | tubulin | small molecule | 2011 (EMA) 2010 (FDA) |
| Ixabepilone | Ixempra | cancer | breast cancer | tubulin | small molecule | 2007 (FDA) no EMA approval |
| Tirbanibulin | Klisyri | cancer | actinic keratosis | SRC Kinase inhibitor tubulin | small molecule | 2021 (EMA) 2020 (FDA) |
| Vinblastin | Velban | cancer | leukemia | tubulin | small molecule | 1965 (FDA) |
| Vincristine | Oncovin | cancer | leukemia small cell lung cancer | tubulin | small molecule | 1963 (FDA) |
| Sirolimus | Rapamune | cancer autoimmune/inflammatory disease | graft rejection kidney transplantation tuberous sclerosis | FK-binding protein 12 | depsipeptide | 2001 (EMA) 1999 (FDA) |
| Everolimus | Afinitor | cancer | renal cell carcinoma breast and pancreatic neoplasms tuberous sclerosis | FK-binding protein 12 | depsipeptide | 2009 (EMA) 2009 (FDA) |
| Tacrolimus | Advagraf | autoimmune/inflammatory disease | atopic dermatitis | FK-binding protein 12 | depsipeptide | 2002 (EMA) 1994 (FDA) |
| Temsirolimus | Torisel | cancer | renal cell carcinoma, cantle-cell lymphoma | FK-binding protein 12 | depsipeptide | 2007 (EMA) 2007 (FDA) |
| [Adalimumab](https://www.drugs.com/mtm/adalimumab.html) | Humira | autoimmune/inflammatory disease | psoriatic, juvenile rheumatoid and rheumatoid arthritis ulcerative colitis ankylosing spondylitis psoriasis crohn’s disease hidradenitis suppurativa | TNF-neutralization | monoclonal antibody | 2003 (EMA) 2002 (FDA) |
| [Etanercept](https://www.drugs.com/mtm/etanercept.html) | Enbrel | autoimmune/inflammatory disease | ankylosing spondylitis psoriatic, juvenile rheumatoid and rheumatoid arthritis  psoriasis | TNF-blocker | recombinant protein | 2000 (EMA) 1998 (FDA) |
| [Infliximab](https://www.drugs.com/infliximab.html) | remicade | autoimmune/inflammatory disease | ankylosing spondylitis psoriatic and rheumatoid arthritis psoriasis crohn’s disease ulcerative colitis | TNF-neutralization | monoclonal antibody | 1999 (EMA) 1998 (FDA) |
| [Golimumab](https://www.drugs.com/mtm/golimumab.html) | Simponi | autoimmune/inflammatory disease | psoriatic and rheumatoid arthritis ankylosing spondylitis ulcerative colitis | TNF-alpha neutralization | monoclonal antibody | 2009 (EMA) 2009 (FDA) |
| [Certolizumab](https://www.drugs.com/mtm/certolizumab.html) | Cimzia | autoimmune/inflammatory disease | rheumatoid arthritis | TNF-alpha neutralization | monoclonal antibody | 2009 (EMA) 2008 (FDA) |
| Prednisonol | Rayos | autoimmune/inflammatory disease | allergic, endocrine, gastrointestinal, neoplastic,  specific infectious, nervous system, ophthalmic,  related organ transplantation, renal, and  rheumatologic conditions; and dermatologic,  hematologic, and pulmonary diseases. | glucocorticoid receptor | small molecule | 1955 (FDA) |
| Methylprednisolone | Medrol | autoimmune/inflammatory disease | endocrine, rheumatic, collagen, dermatologic, allergic, ophthalmic, respiratory, hematologic, neoplastic, edematous, gastrointestinal, nervous system, and other disorders | glucocorticoid receptor | small molecule | 1957 (FDA) before 1995 in EU* |
| Dexamethasone | Decadron | autoimmune/inflammatory disease | inflammatory conditions, including bronchial asthma, as well as endocrine and rheumatic disorders | glucocorticoid receptor | small molecule | 1958 (FDA)  before 1995 in EU* |
| Betamethasone | Diprosone | autoimmune/inflammatory disease | allergic states, dermatologic disorders, gastrointestinal diseases, and hematological disorders | glucocorticoid receptor | small molecule | 1975 (FDA) before 1995 in EU* |
| Hydrocortisone | Cortef | autoimmune/inflammatory disease | endocrine, rheumatic, collagen, allergic, ophthalmic, respiratory, hematologic, neoplastic, edematous, gastrointestinal, and other conditions | glucocorticoid receptor | small molecule | 1952 (FDA) before 1995 in EU* |
| Thalidomide | Thalidomide BMS | cancer | multiple myeloma | cereblon | small molecule | 2008 (EMA)  1998 (FDA) |
| Lenalidomide | Revlimid | cancer | multiple myeloma mantle-cell lymphoma myelodysplastic syndromes | cereblon | small molecule | 2007 (EMA) 2006 (FDA) |
| Lenvatinib | Lenvima | cancer | thyroid neoplasms | VEGFR, FGFR, RET | small molecule | 2015 (EMA) 2015 (FDA) |
| Erlotinib | Tarceva | cancer | pancreatic neoplasms non-small-cell lung carcinoma | EGFR | small molecule | 2005 (EMA) 2004 (FDA) |
| Axitinib | Inlyta | cancer | renal cell carcinoma | VEGFR | small molecule | 2012 (EMA) 2012 (FDA) |
| Avacopan | Tavneos | autoimmune/inflammatory disease | microscopic polyangiitis wegener-granulomatosis | Ca5R | small molecule | 2022 (EMA) 2021 (FDA) |
| Eptifibatide | Integrillin | cardiovascular diseases | unstable angina myocardial infarction | αIIbβ3 integrin | peptide | 1999 (EMA) 1998 (FDA) |
| Abciximab | ReoPro | cardiovascular diseases | myocardial infarction | αIIbβ3 integrin | monoclonal antibody | 1994 (FDA) 1999 (EMA) |
| Tirofiban | Aggrastat | cardiovascular diseases | myocardial infarction | αIIbβ3 integrin | small molecule | 1998 (FDA) |

* initial FDA approval 2004, withdrawn 4 months later

# mutual recognition procedure
